# Enhancement of Biocontrol Efficacy of *Pichia carribbica* to Postharvest Diseases of Strawberries by Addition of Trehalose to the Growth Medium

**DOI:** 10.3390/ijms13033916

**Published:** 2012-03-22

**Authors:** Lina Zhao, Hongyin Zhang, Jun Li, Jinghua Cui, Xiaoyun Zhang, Xiaofeng Ren

**Affiliations:** 1 College of Food and Biological Engineering, Jiangsu University, Zhenjiang 212013, Jiangsu, China; E-Mails: linabobo0706@163.com (L.Z.); zhangxiaoyungu@126.com (X.Z.); renxiaofeng@ujs.edu.cn (X.R); 2 Institute of Life Sciences, Jiangsu University, Zhenjiang 212013, Jiangsu, China; E-Mail: lij_023@163.com; 3 The Library, Jiangsu University, Zhenjiang 212013, Jiangsu, China; E-Mail: cuijing_hua@126.com

**Keywords:** Trehalose, *Pichia caribbica*, postharvest diseases, antagonistic activity, strawberries, differentially expressed proteins

## Abstract

The effects of trehalose on the antagonistic activity of *Pichia caribbica* against *Rhizopus* decay and gray mold decay of strawberries and the possible mechanisms involved were investigated. The proteomic analysis and comparison of *P. carribbica* in response to trehalose was analyzed based on two-dimensional gel electrophoresis. The antagonistic activity of *P. carribbica* harvested from the culture media of NYDB amended with trehalose at 0.5% was improved greatly compared with that without trehalose. The PPO (Polyphenoloxidase) and POD (Peroxidase) activity of strawberries treated with *P. carribbica* cultured in the NYDB media amended with trehalose at 0.5% was higher than that of the strawberries treated with *P. carribbica* harvested from NYDB. The β-1, 3-glucanase activity of strawberries treated with *P. carribbica* cultured in the NYDB media amended with trehalose at 0.5% was also higher than that of the strawberries treated with *P. carribbica* harvested from NYDB and the control. Several differentially expressed proteins of *P. carribbica* in response to trehalose were identified in the cellular proteome, most of them were related to basic metabolism.

## 1. Introduction

The shelf-life of the strawberries is very short because of the postharvest fungal decay, which results in serious economic losses to strawberries. *Rhizopus* decay caused by *Rhizopus stolonifer* (Ehrenb.: Fr) Vuill., and gray mold decay caused by *Botrytis cinerea* Pers.:Fr. are two of the most severe postharvest diseases of strawberries [1,2]. Synthetic fungicides are primarily used to control postharvest decay loss, however, a growing international concern over the often indiscriminate use of synthetic fungicides on food crops because of their possible harmful effects on human health [3]. So an urgent search for alternative control measures with good efficacy, low residues, little or no toxicity to non-target organisms, and no harmful on environment and human health was needed.

Microbial biocontrol agents have shown great potential as an alternative to synthetic fungicides for the control of postharvest decay of fruits and vegetables [4]. Several biological control agents are effective in reducing postharvest decay caused by *Rhizopus stolonifer* and *Botrytis cinerea* on strawberry [5–9]. However, like other non-fungicides means, currently all the biocontrol yeasts cannot reduce postharvest diseases as effectively as synthetic fungicides. So for biological control to be accepted as an economically viable option, the efficacy of antagonistic yeasts in controlling postharvest disease must be enhanced [10].

Many attempts have been investigated to enhance the efficacy of postharvest biocontrol yeasts, including manipulations in the physical and chemical environment during storage, use of mixed cultures, addition of low doses of fungicides in the microbial cultures, addition of salt additives in the microbial cultures, addition of nutrients and plant products in microbial cultures, use of the microbial cultures in association with physical treatments, use of the microbial cultures with other approaches/additives [11]. Besides these attempts above, inducing incubation of the antagonistic yeast has become a useful method to improve the control efficacy of the antagonistic yeast in these years. For example, Yu *et al.* [12] reported that amending with chitin in the culture media may greatly improve the antagonistic activity of *C. laurentii*, and our research team found that the antagonistic activity of *Rhodotorula glutinis* against *Botrytis cinerea* in strawberries could be enhanced by adding chitin in the culture [13].

Trehalose is a unique sugar capable of protecting biomolecules against environmental stress. It is a stable, colorless, odor-free and non-reducing disaccharide, and is widespread in nature. Trehalose has a key role in the survival of some plants and insects, termed anhydrobionts, in harsh environments, even when most of their water body is removed [14]. Trehalose has been found to be optimal in protecting enzymes, antibodies, liposomes and microorganisms, during drying and later storage [15–17]. Combined effects of endo-and exogenous trehalose on stress tolerance and biocontrol efficacy of two antagonistic yeasts was investigated, the result suggested that when adding trehalose in the culture medium, *C. laurentii* and *R. glutinis* resulted in the highest level of biocontrol efficacy against blue mold in apple fruit caused by *Penicillium expansum* Link [18].

*Pichia caribbica* is a strain of antagonistic yeast which was isolated from the soil sample of an unsprayed orchard by our research team, and showed control efficacy to postharvest diseases of apples and pears (unpublished data). However, to our knowledge there is no information concerning enhancement the biocontrol efficacy of *P. caribbica* to postharvest disease of fruits by addition of trehalose to the growth media. In addition to this, most published reports have demonstrated enhancement of biocontrol efficacy of antagonistic yeasts through inducing incubation, but there is limited knowledge of the mode of action.

The objective of this study was to determine the influence of adding trehalose in the culture media on the efficacy of the *P. carribbica* in controlling postharvest *Rhizopus* decay and gray mold decay of strawberries and explore the possible physiological mechanisms involved. Besides, we used 2-DE followed by MALDI-TOF/TOF to investigate and analyze the biological function of the differently expressed proteins between the trehalose inducing incubation *P. carribbica* and the control *P. carribbica* in order to further explain the molecular mechanism of the biological process at proteomic level.

## 2. Results and Discussion

### 2.1. Efficacy of *P. carribbica* Harvested from Different Media in Controlling of *Rhizopus* Decay and Gray Mold Decay of Strawberries

**Figure 1:**
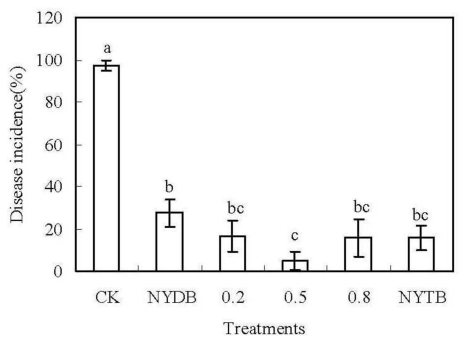
Efficacy of *P. carribbica* harvested from different media in controlling of *Rhizopus* decay of strawberries. Fruit treatments are as follows: sterile distilled water (CK), *P. carribbica* harvested from the media of NYDB (NYDB), *P. carribbica* harvested from NYDB amended with trehalose at 0.2% (0.2), *P. carribbica* harvested from NYDB amended with trehalose at 0.5% (0.5), *P. carribbica* harvested from NYDB amended with trehalose at 0.8% (0.8), *P. carribbica* harvested from NYTB (NYTB). Bars represent standard errors. Data in columns with different letters are statistically different according to Duncan’s multiple range test at *P* = 0.05.

All treatments with application of *P. carribbica* at 1 × 10^8^ cells/mL could reduce the disease incidence of *Rhizopus* decay of strawberries, compared with the control after 3 days of incubation at 20 °C (*P* < 0.05) (Figure 1). The antagonistic activity of *P. carribbica* was shown to be greatly enhanced through cultured in the NYDB media amended with 0.5% trehalose. The disease incidence of *Rhizopus* decay of strawberries treated with *P. carribbica* which were harvested from the media of NYDB amended with trehalose powder at 0.5% was 5%, which was significantly lower than that of the control strawberries (97.5%) and the strawberries treated with *P. carribbica* which were harvested from the media of NYDB (27.5%). However, the antagonistic activity of *P. carribbica* was not enhanced through being incubated in the NYDB media amended with 0.2% or 0.8% trehalose, or NYTB media, compared with that incubated in NYDB without trehalose.

**Figure 2:**
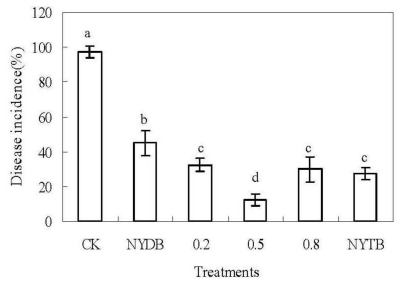
Efficacy of *P. carribbica* harvested from different media in controlling of gray mold decay of strawberries. Fruit treatments are as follows: sterile distilled water (CK), *P. carribbica* harvested from the media of NYDB (NYDB), *P. carribbica* harvested from NYDB amended with trehalose at 0.2% (0.2), *P. carribbica* harvested from NYDB amended with trehalose at 0.5% (0.5), *P. carribbica* harvested from NYDB amended with trehalose at 0.8% (0.8), *P. carribbica* harvested from NYTB (NYTB). Bars represent standard errors. Data in columns with different letters are statistically different according to Duncan’s multiple range test at *P* = 0.05.

All treatments with application of *P. carribbica* at 1 × 10^8^ cells/mL could reduce the disease incidence of gray mold decay of strawberries, compared with the control after 3 days of incubation at 20 °C (*P* < 0.05) (Figure 2). The disease incidence of gray mold decay of strawberries treated with *P. carribbica* which were harvested from the media of NYDB amended with trehalose at 0.2%, 0.5%, 0.8% or 1% was significantly lower than the strawberries treated with *P. carribbica* which were harvested from the media of NYDB (45%), especially when amended with trehalose at 0.5% (12.5%).

These results showed that *P. carribbica* has control efficacy to postharvest *Rhizopus* decay and gray mold decay of strawberries. Besides, the biological control activity of *P. carribbica* against *Rhizopus* decay and gray mold decay of strawberries was greatly enhanced when the yeast was harvested from the culture media of NYDB amended with 0.5% trehalose, compared with the case that *P. carribbica* harvested from NYDB without trehalose. Since that trehalose has no risks to humans and to the environment (it has been approved as a health food by FDA), and many tests approved that trehalose is non-toxicity, besides trehalose is a cheap and widely available natural resource [19], the utilization of trehalose might be an effective, safe and economic approach to enhance the biocontrol efficacy of *P. carribbica*.

### 2.2. Effects of *P. carribbica* Harvested from Different Media on PPO, POD and β-1,3-glucanase Activities of Strawberries

**Figure 3:**
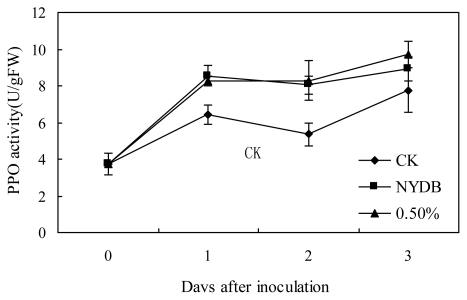
Effects of *P. carribbica* harvested from different media on PPO activity of strawberries. Fruit treatments are as follows: sterile distilled water (CK), *P. carribbica* harvested from the media of NYDB (NYDB), and *P. carribbica* harvested from NYDB amended with trehalose at 0.5% (0.5%). Bars represent standard errors.

The PPO activities of strawberries treated with *P. carribbica* harvested from NYDB or NYDB amended with trehalose at 0.5% or the control increased gradually, and then began to decrease (Figure 3). The PPO activities of strawberries treated with *P. carribbica* harvested from NYDB or NYDB amended with trehalose at 0.5% were higher than that of the control at all the storage time. There was no significant difference between the PPO activity of strawberries treated with *P. carribbica* harvested from NYDB amended with trehalose at 0.5% and that of the strawberries treated with *P. carribbica* harvested from NYDB without trehalose at 1 and 2 days. However, the PPO activity of strawberries treated with *P. carribbica* harvested from NYDB amended with trehalose at 0.5% was higher than that of the strawberries treated with *P. carribbica* harvested from NYDB without trehalose at 3 days.

**Figure 4:**
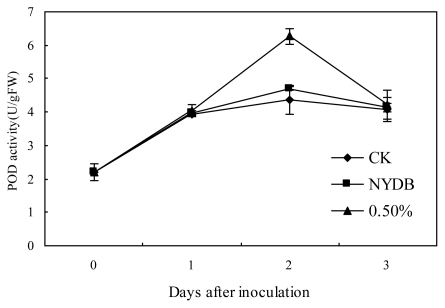
Effects of *P. carribbica* harvested from different media on POD activity of strawberries. Fruit treatments are as follows: sterile distilled water (CK), *P. carribbica* harvested from the media of NYDB (NYDB), and *P. carribbica* harvested from NYDB amended with trehalose at 0.5% (0.5%). Bars represent standard errors.

The POD activities of strawberries treated with *P. carribbica* harvested from NYDB or NYDB amended with trehalose at 0.5% or the control gradually increased, the POD activity reached the highest peak at 2 days, and then began to decrease at 3 days (Figure 4). The POD activity of strawberries treated with *P. carribbica* either cultured in NYDB media amended with trehalose at 0.5% or cultured in NYDB without trehalose were higher than that of the control at 2 days after storage, and the POD activity of the strawberries treated with *P. carribbica* cultured in the NYDB media amended with trehalose at 0.5% were higher than that of the strawberries treated with *P. carribbica* cultured in NYDB without trehalose at 2 days after storage.

**Figure 5:**
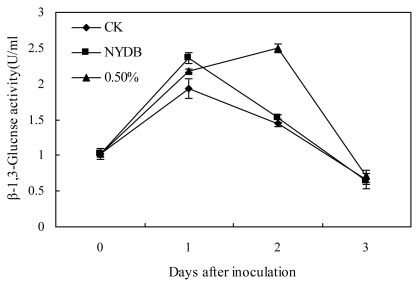
Effects of *P. carribbica* harvested from different media on β-1,3-glucanase activity of strawberries. Fruit treatments are as follows: sterile distilled water (CK), *P. carribbica* harvested from the media of NYDB (NYDB), and *P. carribbica* harvested from NYDB amended with trehalose at 0.5% (0.5%). Bars represent standard errors.

The β-1,3-glucanase activity of strawberries treated with *P. carribbica* harvested from NYDB and water increased quickly at the first day, and began to decrease at 2 days (Figure 5). However, the β-1, 3-glucanase activity of strawberries treated with *P. carribbica* harvested from NYDB amended with trehalose at 0.5% increased quickly at the first day and the second day, and began to decrease after 2 days. The β-1,3-glucanase activities of strawberries treated with *P. carribbica* harvested from NYDB and NYDB amended with trehalose at 0.5% were higher than that of the control strawberries at 1 day, and the β-1,3-glucanase activities of strawberries treated with *P. carribbica* harvested from NYDB amended with trehalose at 0.5% was higher than that of the control strawberries and the strawberries treated with *P. carribbica* harvested from NYDB at 2 days.

Polyphenoloxidase (PPO) catalyzes the oxidation of phenolics to quinines, which are more toxic to pathogens than the former [20]. Increased PPO activity is correlated with disease resistance in plants [21,22]. Peroxidases (POD) as bifunctional enzymes, can oxidize various substrates in the presence of H_2_O_2_, but also produce reactive oxygen species [23]. High POD activity is associated with the onset of induced resistance, which involves in several plant defence mechanisms, such as lignification and suberization [24,25]. The results showed that the PPO activity of strawberries treated with *P. carribbica* either cultured in the NYDB media amended with trehalose at 0.5% or cultured in NYDB without trehalose were higher than that of the control at all the storage time, and the POD activity of strawberries treated with *P. carribbica* either cultured in the NYDB media amended with trehalose at 0.5% or cultured in NYDB without trehalose were higher than that of the control at some storage time. What’s more, the PPO and POD activity of strawberries treated with *P. carribbica* cultured in the NYDB media amended with trehalose at 0.5% was higher than that of strawberries treated with *P. carribbica* harvested from NYDB at some of the storage time. These results suggest that treatment of *P. carribbica* can enhance lignification and suberization of strawberries, which are related to maintenance of integrity and vital functions of the cell, and increase the levels of antimicrobial phenolic compounds, so that result in improvement of disease resistance. Moreover, adding 0.5% trehalose in the media can enhance this activity of *P. carribbica* to improve disease resistance of fruits. This may be one mechanism by which adding trehalose in the culture medium enhances the biocontrol efficacy of *P. carribbica* to postharvest *Rhizopus* decay and gray mold decay of strawberries.

Our study displayed that *P. carribbica* either cultured in the NYDB media amended with trehalose at 0.5% or cultured in NYDB without trehalose induced more β-1,3-glucanase activity of strawberries compared with the control at some of the storage time, and *P. carribbica* cultivated in NYDB media amended with trehalose at 0.5% induced more β-1,3-glucanase activity of strawberries compared with *P. carribbica* cultured in the NYDB at some of the storage time. A previous study indicates that in plants, invasion by a pathogen induces the production of pothogenesis-related (PR) proteins, such as β-1,3-glucanases, and the putative role of β-1,3-glucanases in disease resistance is related to their capacity to degrade fungal cell wall, mainly composed of β-1,3-glucan [26]. This induction can be enhanced by some elicitors with the improvement of resistance to pathogens in plants [27,28]. Therefore, we infer that improving the activity of antagonistic yeast to induce the production of pothogenesis-related (PR) proteins of strawberries may be another reason that a more remarkable upward trend was seen in the efficacy of *P. carribbica* cultivated in the NYDB media amended with 0.5% trehalose in controlling postharvest decay of strawberries than the case in NYDB.

### 2.3. Identification of Differentially Expressed Proteins

**Figure 6:**
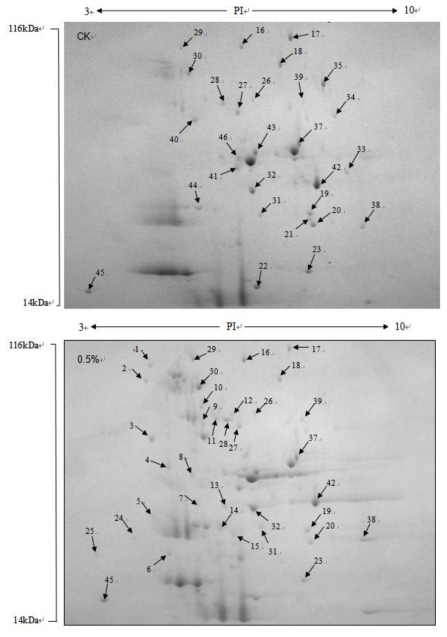
Two-dimensional pattern of intracellular proteins of *P. carribbica* after cultivation in NYDB or NYDB amended with 0.5% trehalose powder.

For protein identification by means of peptide mass fingerprints (PMF), we used MASCOT to search protein database of Viridiplantae. More than 180 protein spots were detected in each gel after ignoring very faint spots and spots with undefined shapes and areas using Image Master 2D Elite software (Figure 6). A total of 78 proteins were differentially expressed when the yeast antagonist *P. carribbica* were harvested from the NYDB amended with trehalose at 0.5%. Of the 78 proteins, 53 proteins were up-regulated and 25 proteins were down-regulated. Our test focuses on 46 kinds of differences in degree of peak protein (Table 1). Of all the differentially expressed proteins identified, most of them related to basic metabolism. This indicated that the basic metabolism of *P. carribbica* was improved by trehalose induced incubation, so as to improve its biocontrol efficacy to postharvest diseases of strawberries. Response patterns of *P. carribbica* to trehalose inducing incubation are complex, as the differentially abundant proteins are involved in multiple metabolic pathways.

## 3. Experimental Section

### 3.1. Antagonist and Growth Conditions

The yeast antagonist *P. carribbica* was isolated from soils of orchard (the central shoal of Yangtze River, Zhenjiang). Classical methods based on colony and cell morphologies were used for a preliminary characterization of the yeast [29]. Subsequently, sequence analysis of the 5.8S internal transcribed spacer (ITS) ribosomal DNA (rDNA) region was used to identify the yeast colony [30]. *P. carribbica* isolates were maintained at 4 °C on nutrient yeast dextrose agar (NYDA) medium containing 8 g nutrient broth, 5 g yeast extract, 10 g glucose and 20 g agar, in 1 L of distilled water. Liquid cultures of the yeast were grown in 250-mL Erlenmeyer flasks containing 50 mL of nutrient yeast dextrose broth (NYDB) which had been inoculated with a loop of the culture. Flasks were incubated on a rotary shaker at 180 rpm at 28 °C for 24 h. Following incubation, cells were centrifuged at 6000 ×*g* for 10 min and washed twice with sterile distilled water in order to remove the growth medium. Cell pellets were re-suspended in sterile distilled water and adjusted to an initial concentration of 5 × 10^8^ cells/mL. Then, 1 mL of the above-mentioned suspensions were added and cultivated in nutrient yeast dextrose broth (NYDB) or NYDB amended with trehalose powder at 0.2% (using 0.8% dextrose and 0.2% trehalose as the carbon source) or 0.5% (using 0.5% dextrose and 0.5% trehalose as the carbon source) or 0.8% (using 0.2% dextrose and 0.8% trehalose as the carbon source) or NYTB (trehalose as the sole carbon source instead of dextrose in the media of nutrient yeast dextrose broth) on a rotary shaker at 180 rpm at 28 °C for 24 h. Then the yeast cells were harvested by centrifuging at 6000 ×*g* for 10 min and were washed twice with sterile distilled water. The yeast cell was counted using a hemocytometer. Cell pellets were re-suspended in sterile distilled water and adjusted to an initial concentration of 1 × 10^8^ cells/mL for experiments.

### 3.2. Fruits

Strawberries (*Fragaria ananassa* Duch.) cultivars “fengxiang” were harvested early in the morning and rapidly transferred to the laboratory. Berries were sorted on the basis of size, ripeness and to remove any with the apparent injuries or infections.

### 3.3. Pathogen Inoculum

The pathogen *Rhizopus stolonifer* (Ehrenb.: Fr) Vuill. and *Botrytis cinerea* (Pers.:Fr.) were isolated from infected strawberries. The culture was maintained on potato dextrose agar (PDA: extract of boiled potatoes, 200 mL; dextrose, 20 g; agar, 20 g and distilled water, 800 mL) at 4 °C, and fresh cultures were grown on PDA plates before use. Spore suspensions were prepared by removing the spores from the sporulating edges of a 7-day old culture with a bacteriological loop, and suspending them in sterile distilled water. Spore concentrations were determined with a hemocytometer, and adjusted as required with sterile distilled water.

### 3.4. Efficacy of *P. carribbica* Harvested from Different Media in Controlling of *Rhizopus* Decay and Gray Mold Decay of Strawberries

The surface of strawberries was wounded with a sterile cork borer (approximately 3-mm-diameter and 3-mm-deep) and treated with 30 μL of (1) the cell suspensions of *P. carribbica* (1 × 10^8^ cells/mL) which were harvested from the media of NYDB; (2) the cell suspensions of *P. carribbica* (1 × 10^8^ cells/mL) which were harvested from the media of NYDB amended with trehalose powder at 0.2%; (3) the cell suspensions of *P. carribbica* (1 × 10^8^ cells/mL) which were harvested from the media of NYDB amended with trehalose powder at 0.5%; (4) the cell suspensions of *P. carribbica* (1×10^8^ cells/mL) which were harvested from the media of NYDB amended with trehalose powder at 0.8%; (5) the cell suspensions of *P. carribbica* (1 × 10^8^ cells/mL) which were harvested from the media of NYTB and (6) the sterile distilled water as the control. Three hours later, 30 μL of *R. stolonifer* suspensions (1 × 10^4^ spores/mL) or *B. cinerea* suspensions (1 × 10^5^ spores/mL) were inoculated to each wound. After air-drying, the strawberries were stored in enclosed plastic trays to maintain a high relative humidity (about 95%) and incubated at 20 °C for 3 days. The number of the infected fruit wounds was examined. There were three replicates per treatment and 20 fruits each replicate. All treatments were arranged in a randomized complete block design, and the experiment was conducted twice.

### 3.5. Effects of *P. carribbica* Harvested from Different Media on PPO (Polyphenoloxidase), POD (Peroxidase) and β-1,3-glucanase Activities of Strawberries

The surface of strawberries was wounded with a sterile cork borer (approximately 3-mm-diameter and 3-mm-deep) and treated with 30 μL of a cell suspension of *P. carribbica* (1 × 10^8^ cells/mL) which were harvested from the media of NYDB or NYDB amended with trehalose powder at 0.5% (using 0.5% dextrose and 0.5% trehalose as the carbon source) after 24 h incubation. Treatment with sterile distilled water served as the control. After air-drying, the strawberries were stored in enclosed plastic trays to maintain a high relative humidity (about 95%) and incubated at 20 °C. Samples were taken at 0, 1, 2, and 3 days after treatment. After removing the wound tissue with a sterile borer (6-mm-diameter and 5-mm-deep), the fresh tissue around the wound was picked up by another sterile borer (9-mm-diameter and 10-mm-deep). Two grams of the fresh tissue from six fruits were homogenized with 10 mL of cold (4 °C) 50 mM (pH 7.8) sodium phosphate buffer containing 1.33 mM EDTA and 1% PVPP. The homogenates were then centrifuged at 12,000 ×*g* at 4 °C for 15 min and the supernatants were assayed. There were three replicates per treatment. The experiment was conducted twice. The testing methods of enzyme activities were described below.

PPO activity was measured as the method described by Aquino-Bolaños and Mercado-Silva [31] with some modifications, using catechol as a substrate. The reaction mixtures contained 2.9 mL of 0.1 M catechol (prepared by 50 mM sodium phosphate buffer, pH 6.4, incubated at 30 °C for 5 min) as a substrate and 100 μL of enzymatic extract. The change in absorbance was read per minute at 398 nm, and determined 3 min continuously. The PPO activity was expressed as U per g of fresh tissue weight (U/g FW), which one unit (U) of PPO was defined as the change of absorbance ascent 0.01 in 1 min.

POD activity was measured as the method described by Lurie *et al.* [24] with some modifications, using guaiacol as a substrate. The reaction mixture contained 0.2 mL of crude enzyme extract (supernatant extract), 2.2 mL of 0.3% guaiacol (prepared by 50 mM sodium phosphate buffer, pH 6.4) and was incubated for 5 min at 30 °C. The reaction was then initiated immediately by adding 0.6 mL of 0.3% H_2_O_2_ (prepared by 50 mM sodium phosphate, pH 6.4, and incubated at 30 °C for 5 min) and the activity was determined by measuring at A_470_ once every 1 min for 3 min. A cuvette containing all components except adding 0.6 mL of distilled water was used as a control. The POD activity was expressed as U per g fresh tissue weight (U/g FW). One unit was defined as an increase in A _470_ of 0.01 per minute.

β-1,3-glucanase was assayed by measuring the amount of reducing sugar released from the substrate by the method reported by Ippolito *et al.* [32] with some modifications. Crude enzyme sample of 250 μL was routinely added to 250 μL of 0.2% laminarin (*w*/*v*) in 50 mM, pH 5.0, and potassium acetate buffer and incubated at 37 °C for 1 h. For the control, the same mixture was similarly diluted at zero incubation time. The reaction was stopped by adding 1.5 mL of 3, 5-dinitro-salicylate and boiling for 5 min on a water bath. And the amount of reducing sugars was measured spectrophotometrically at 500 nm using a UV-1601 spectrophotometer (Shimadzu, Japan). One unit of the β-1,3-glucanase was defined as the formation of 1 mg glucose equivalents per hour and the specific activity was expressed as the U per gram FW.

### 3.6. Protein Sample Preparation

Liquid cultures of the yeast were grown in NYDB or NYDB+ 0.5% trehalose, respectively as described above (Antagonist and growth conditions). The method of protein sample preparation was described by Li *et al.* [33] with some modifications. The yeast cells were harvested from NYDB or NYDB+ 0.5% trehalose by centrifuging at 10,000 ×*g* for 10 min (4°C) and washed three times with cold double-distilled water to remove residual medium. Then grind sample (yeast cells) into a fine powder in a mortar pestle under liquid nitrogen, a fine powder is important for effective contaminant removal and protein extraction. Transfer the powder into a 10-mL tube, filled the tube with TE buffer containing 10 mM Tris-HCL, pH 8.0, 1 mM EDTA, and 1 mM PMSF, incubated at 4 °C for 30 min. Mixed well by vortexing, and take 0.5 mL to 1.5 mL EP tube, added 8.7 μL of 10 mg mL^−1^ PMSF and several 0.5 mm glass beads, vortex oscillator 5 × 30 s, and interval ice bath for 1 min. Then added 25 μg of RNase A and 100 μg of DNase, incubated at 4 °C for 30 min. Centrifuge at 15,000 ×*g* for 30 min (4 °C), take the supernatant 400 μL, added three volumes of 20% TCA/acetone (−20 °C pre-cooled at least 30 min), mixed well, incubated at −20 °C for 12–16 h. Centrifuge at 15,000 ×*g* for 30 min (4 °C), discard the supernatant, washed with acetone (−20 °C pre-cooled) several times, and after washed every time, centrifuge at 15,000 ×*g* for 30 min (4 °C), and discard the supernatant. Air-dry at room temperature at least 10 min to remove residual acetone. Then solubilized in 100 μL of thiourea/urea/lysis buffer containing 2 M thiourea, 7 M urea, 4% (*w*/*v*) CHAPS, 65 mM DTT, 0.2% (*w*/*v*) Bio-Lyte. Protein samples were kept at −70°C until use. The protein concentration was determined according to Bradford’s method using bovine serum albumin as standard [34].

### 3.7. 2-DE and Image Analysis

Two-dimensional electrophoresis (2-DE) and image analysis were performed as described by Wang *et al.* [35]. 380 μg of each protein extract was separated by iso-electrophoresis using IPG strips (pH 3–10, 17 cm) in the Protean system (Bio-Rad). The second dimension was run on a 12.5% polyacrylamide gel using the Multiphor system (Amersham Biosciences). Gels were visualized by Coomassie Blue staining. The stained gels were scanned and analyzed using PDQuest software (version 7.2, Bio-Rad, Hercules, CA, USA). Proteins that were increased 2-fold at least at one point after treatment, as well as exhibiting the same expression pattern among the replicates, were considered as significant and reproducible changes proteins. These proteins were subjected to identification. At least three biological replicates were performed for each treatment.

### 3.8. Protein In-gel Digestion

Differentially expressed protein spots were excised from the gel and were washed twice by double-distilled H_2_O, then destained with 50 mM NH_4_CO_3_/acetonitrile solution. Washed with 25 mM NH_4_CO_3_, 50% acetonitrile and acetonitrile until the gels became full white, then vacuum drained 5 min. Added 2 μL 10 μg·μL^−1^ trypsin (Sigma–Aldrich, St Louis, MO, USA), incubated at 4 °C for 30 min, then added 10 μL 25 mM NH_4_CO_3_, the gels were incubated overnight at 37 °C. The supernatant was collected for MS analysis [36].

### 3.9. Protein Identification by MALDI-TOF/TOF and Database Query

The peptide solution was analyzed using MALDI TOF/TOF mass spectrometer (Ultraflex III, Bruker-Daltonics). The resulting monoisotopic peptide masses were queried against protein database in NCBInr using MASCOT software [37] and the following search parameters: all entries, trypsin, up to one missed cleavage, carbamidomethyl(C), oxidation(M) and Gln-Pyro-glu, peptide tolerance 0.3 Dal, mass value MH^+^, and monoisotopic [38].

### 3.10. Statistical Analysis

The data were analyzed by the analysis of variance (ANOVA) in the statistical program SPSS/PC version II.x, (SPSS Inc. Chicago, Illinois, USA) and the Duncan’s multiple range test was used for means separation. In addition, when the group of the data was two, the independent samples t test was applied for means separation. The statistical significance was assessed at the level *P* = 0.05.

## 4. Conclusions

Our results showed that the antagonistic activity of *P. carribbica* to postharvest *Rhizopus* decay and gray mold decay of strawberries can be enhanced by adding trehalose in the media, which may offer great practical potential in reducing the postharvest diseases of strawberries. The mode of action may be involved in trehalose inducing incubation *P. carribbica* induce more defense enzymes such as PPO, POD, and pothogenesis-related (PR) proteins such as β-1,3-glucanase of fruits, to improve disease resistance of strawberries. Identification of differentially expressed proteins showed the basic metabolism of *P. carribbica* was improved by trehalose induced incubation. These mechanisms need further study.

## Figures and Tables

**Table 1:** Identification of cellular proteins of *P. carribbica* showing differential expression under trehalose cultivation using MS/MS analysis.

Spot	Protein name	NCBI accession	Mass	PI	Species	Score
1	50S ribosomal protein	gi|116495724	12511	4.54	*Lactobacillus casei* ATCC 334	66
2	UDP-galactopyranose mutase	gi|326332321	45474	5.11	*Nocardioidaceae bacterium* Broad-1	68
3	extracellular solute-binding protein	gi|148546296	45335	6.02	*Pseudomonas putida* F1	62
4	eukaryotic initiation factor 4A	gi|146422477	44615	4.91	*Meyerozyma guilliermondii* ATCC 6260	100
5	predicted protein	gi|145345294	42354	9.84	*Ostreococcus lucimarinus* CCE9901	65
6	hypothetical protein SCHCODRAFT_38806	gi|302688397	10646	4.25	*Schizophyllum commune* H4-8	51
8	elongation factor Tu	gi|587590	43823	5.06	*Wolinella succinogenes*	75
9	electron transfer flavoprotein subunit beta	gi|162447962	29409	8.87	*Acholeplasma laidlawii* PG-8A	69
10	hypothetical protein bthur0013_57560	gi|228911633	45801	6.33	*Bacillus thuringiensis* IBL 200	62
11	HNH nuclease	gi|220919264	38869	9.73	*Anaeromyxobacter dehalogenans* 2CP-1	65
12	hypothetical protein PGUG_04322	gi|146415246	69936	5.30	*Meyerozyma guilliermondii* ATCC 6260	71
13	hypothetical protein bcere0017_55820	gi|229119349	28408	8.55	*Bacillus cereus* Rock1-3	67
14	hypothetical protein PGUG_00294	gi|146421948	35820	5.22	*Meyerozyma guilliermondii* ATCC 6260	104
15	xylose reductase	gi|4103055	36076	5.58	*Meyerozyma guilliermondii*	66
16	conserved hypothetical protein	gi|146421560	84405	5.92	*Meyerozyma guilliermondii* ATCC 6260	90
17	conserved hypothetical protein	gi|146420955	85681	5.68	*Meyerozyma guilliermondii* ATCC 6260	134
18	hypothetical protein CLOSCI_01190	gi|167758847	66473	4.76	*Clostridium scindens* ATCC 35704	68
19	translation elongation factor	gi|47176804	22272	5.46	*Meyerozyma guilliermondii*	68
20	conserved hypothetical protein	gi|146417765	34916	7.17	*Meyerozyma guilliermondii* ATCC 6260	96
21	translation elongation factor	gi|47176804	22272	5.46	*Meyerozyma guilliermondii*	82
22	Melibiase subfamily, putative	gi|254503502	77433	5.48	*Labrenzia alexandrii* DFL-11	86
24	hypothetical protein PGUG_05024	gi|146414197	32004	7.77	*Meyerozyma guilliermondii* ATCC 6260	80
25	unnamed protein product	gi|189054178	65980	7.62	*Homo sapiens*	87
26	hypothetical protein	gi|67601196	32428	9.67	*Cryptosporidium hominis* TU502	63
27	hypothetical protein PGUG_02894	gi|146418399	57751	5.57	*Meyerozyma guilliermondii* ATCC 6260	89
28	transcriptional regulator, laci family	gi|315498361	34890	5.36	*Asticcacaulis excentricus* CB 48	87

29	nitrite and sulfite reductase 4Fe-4S region	gi|118579082	23982	8.66	*Pelobacter propionicus* DSM 2379	71
30	heat shock protein SSB1	gi|146420661	66250	5.29	*Meyerozyma guilliermondii* ATCC 6260	162
31	cytochrome d ubiquinol oxidase subunit III	gi|156973199	16378	4.72	*Vibrio harveyi* ATCC BAA-1116	71
32	FAD dependent oxidoreductase	gi|225011369	41131	8.66	*Flavobacteria bacterium* MS024-2A	73
33	PREDICTED: uncharacterized glycosyltransferase AER61- like	gi|109036798	62132	6.39	*Macaca mulatta*	61
34	hypothetical protein KSE_48990	gi|311898269	37649	8.94	*Kitasatospora setae* KM-6054	48
35	isocitrate lyase	gi|146413757	61766	6.31	*Meyerozyma guilliermondii* ATCC 6260	88
36	conserved hypothetical protein	gi|238064394	46346	11.42	*Micromonospora sp.* ATCC 39149	78
37	DEHA2D06160p	gi|50420381	54282	5.68	*Debaryomyces hansenii* CBS767	72
38	glyceraldehyde-3-phosphate dehydrogenase	gi|146419367	35717	6.60	*Meyerozyma guilliermondii* ATCC 6260	82
39	hypothetical protein HMU03290	gi|291276562	8800	9.70	*Helicobacter mustelae* 12198	67
40	conserved hypothetical protein	gi|313836798	12685	6.31	*Propionibacterium acnes* HL037PA2 78	
41	possible gp16 protein	gi|227496471	16688	4.70	*Actinomyces urogenitalis* DSM 15434	79
42	conserved hypothetical protein	gi|301168105	15154	9.27	*Bacteriovorax marinus* SJ	82
43	enolase 1	gi|146415384	46951	5.42	*Meyerozyma guilliermondii* ATCC 6260	84
44	rod shape-determining protein MreC, putative	gi|21673403	32080	9.73	*Chlorobium tepidum* TLS	86
45	hypothetical protein LVIS_1868	gi|116334433	7652	5.55	*Lactobacillus brevis* ATCC 367	85
46	enolase 1	gi|146415384	46951	5.42	*Meyerozyma guilliermondii* ATCC 6260	97
